# Deciphering the human antibody response against *Burkholderia pseudomallei* during melioidosis using a comprehensive immunoproteome approach

**DOI:** 10.3389/fimmu.2023.1294113

**Published:** 2023-12-11

**Authors:** Gabriel E. Wagner, Thomas Franz Paul Stanjek, Dirk Albrecht, Michaela Lipp, Susanna J. Dunachie, Esther Föderl-Höbenreich, Katharina Riedel, Anne Kohler, Ivo Steinmetz, Christian Kohler

**Affiliations:** ^1^ Diagnostic and Research Institute of Hygiene, Microbiology and Environmental Medicine, Medical University of Graz, Graz, Austria; ^2^ Friedrich Loeffler Institute of Medical Microbiology, University Medicine, Greifswald, Germany; ^3^ Institute of Microbiology, Department of Microbial Physiology and Molecular Biology, University of Greifswald, Greifswald, Germany; ^4^ Nuffield Department of Medicine (NDM) Centre for Global Health Research, Nuffield Department of Medicine, University of Oxford, Oxford, United Kingdom; ^5^ National Institute for Health and Care Research (NIHR) Oxford Biomedical Centre, Oxford University Hospitals National Health Service (NHS) Foundation Trust, Oxford, United Kingdom; ^6^ Mahidol-Oxford Tropical Medicine Research Unit, Mahidol University, Bangkok, Thailand; ^7^ Diagnostic & Research Institute of Pathology, Medical University Graz, Graz, Austria

**Keywords:** *Burkholderia pseudomallei*, immunoproteomics, melioidosis, dot blot, Western blot

## Abstract

**Introduction:**

The environmental bacterium *Burkholderia pseudomallei* causes the often fatal and massively underreported infectious disease melioidosis. Antigens inducing protective immunity in experimental models have recently been identified and serodiagnostic tools have been improved. However, further elucidation of the antigenic repertoire of *B. pseudomallei* during human infection for diagnostic and vaccine purposes is required. The adaptation of *B. pseudomallei* to very different habitats is reflected by a huge genome and a selective transcriptional response to a variety of conditions. We, therefore, hypothesized that exposure of *B. pseudomallei* to culture conditions mimicking habitats encountered in the human host might unravel novel antigens that are recognized by melioidosis patients.

**Methods and results:**

In this study, *B. pseudomallei* was exposed to various stress and growth conditions, including anaerobiosis, acid stress, oxidative stress, iron starvation and osmotic stress. Immunogenic proteins were identified by probing two-dimensional Western blots of *B. pseudomallei* intracellular and extracellular protein extracts with sera from melioidosis patients and controls and subsequent MALDI-TOF MS. Among *B. pseudomallei* specific immunogenic signals, 90 % (55/61) of extracellular immunogenic proteins were identified by acid, osmotic or oxidative stress. A total of 84 % (44/52) of intracellular antigens originated from the stationary growth phase, acidic, oxidative and anaerobic conditions. The majority of the extracellular and intracellular protein antigens were identified in only one of the various stress conditions. Sixty-three immunoreactive proteins and an additional 38 candidates from a literature screening were heterologously expressed and subjected to dot blot analysis using melioidosis sera and controls. Our experiments confirmed melioidosis-specific signals in 58 of our immunoproteome candidates. These include 15 antigens with average signal ratios (melioidosis:controls) greater than 10 and another 26 with average ratios greater than 5, including new promising serodiagnostic candidates with a very high signal-to-noise ratio.

**Conclusion:**

Our study shows that a comprehensive *B. pseudomallei* immunoproteomics approach, using conditions which are likely to be encountered during infection, can identify novel antibody targets previously unrecognized in human melioidosis.

## Introduction

The Gram-negative environmental bacterium *Burkholderia pseudomallei*, endemic in Southeast Asia and northern Australia, causes the often fatal tropical infectious disease melioidosis. The latter is known as the great mimicker, as clinical manifestations are extremely variable and unspecific with pneumonia and sepsis being the most common clinical presentations (1–5). Severe melioidosis with bacteremia is associated with a high case fatality rate still reaching 40% (6). With 165,000 cases of human melioidosis per year worldwide, from which 89,000 patients die, as predicted by Limmathurotsakul (2), it falls between measles and leptospirosis (6). This not only demonstrates the high burden of the disease (7) but also the importance of unraveling its true global prevalence (e.g. by seroprevalence studies). There is even more demand for methods that reliably detect exposure and infection because *B. pseudomallei* is a Tier 1 select agent and potential bioweapon.

Surveillance and diagnosis are complicated by the lack of awareness and microbiological capacity in many predicted endemic areas (1, 4, 8) and issues related to diagnostic tests. The diagnostic gold standard, the cultural detection of the pathogen, not only requires special expertise and strict laboratory safety procedures, but also lacks sensitivity, and the time to result is not fast enough for many clinical settings (9). Other tests available for direct pathogen detection, such as antigen tests or quantitative real-time PCR, also have limitations due to low sensitivities or costly laboratory equipment and reagents, which are not available in many endemic areas (6, 9, 10). Serodiagnostic methods show great promise in *B. pseudomallei* diagnostics because of their cost, ease of use and timely test results. In the past, those methods using crude antigen preparations were hampered by low sensitivity (roughly 56%) and high background seropositivity in endemic areas (6, 11, 12). Serological melioidosis assays [e.g. microarrays (13, 14), ELISA (15), lateral/vertical flow (16, 17)] relying on purified *B. pseudomallei* antigens have attracted attention recently due to enhanced performance (13, 16, 18, 19). Although the sensitivities and specificities of these assays in the diagnosis of acute melioidosis on admission have improved compared to standard methods, it is noteworthy that there are still issues to be addressed. Exemplarily, 8% of the melioidosis patients did not produce detectable antibodies against the serodiagnostic antigens available in our previous study (13). In the case of the hemolysin coregulated protein 1 (Hcp1), currently the best performing serodiagnostic antigen (16, 19), variants with lower antigenicity have been identified (20). It has also been reported that a limitation of a singleplex Hcp1 assay is that it cannot distinguish between a past and present infection (21). The issue might be addressed by the use of multiplex assays. In such cases, additional antigens might serve as serodiagnostic backup and/or increase the sensitivity and reliability of the assays (13, 19). However, compared to the analysis of the innate immune response in melioidosis (22–24), the literature on the antibody response, particularly to distinct *B. pseudomallei* protein antigens, is scarce and incomprehensive (25–29) despite the implications for diagnostics and vaccine development. Here, information-dense microarrays, which enable the screening of dozens to hundreds of analytes in parallel, offer the possibility of performing large-scale serological surveillance studies and serodiagnostics at unpaired resolution (13, 28, 30). In addition to the simple analysis of the response regarding a single antigen, these highly multiplexed tests allow the comparison of entire antibody response profiles. It will be very interesting to analyze whether there are certain profiles associated with, for example, acute and chronic disease, reactivation, latent infection and protective immunity. Furthermore, there are known cross-reactivities between the antibody response to *B. pseudomallei* and other *Burkholderia* species of lower pathogenicity (31, 32) that might also be resolved based on the associated profiles. Since there is no vaccine available, such analyses and the detailed insights into the (protective) immune response of melioidosis patients may also lead to the identification of additional antigenic candidates for subunit vaccines.

We present here a novel approach to identify additional protein antigens addressing the aforementioned need for additional serodiagnostic biomarkers and show that these biomarkers enable a more detailed picture of the immune response during melioidosis. We exploited the fact that *B. pseudomallei* can easily adapt to a variety of habitats and hosts due to its comparatively large genome [2 chromosomes, 7.2 megabase pairs, 6000 to 6800 coding sequences (33–36)]. While a typical immunoproteomic approach relies on cultures grown under (a few) standard conditions, we hypothesized that novel antigens could be more comprehensively identified if the bacteria were grown under stress conditions similar to those found in the human host. Indeed, these stressors led to a shift in the proteome that is not observed under standard conditions and significantly increased the antigenic repertoire as determined by two-dimensional (2D) immunoproteomics. Further validation of the most promising candidates using recombinantly expressed proteins led to the identification of novel, previously unknown antigens with diagnostic potential.

## Materials and methods

### Ethics statement

Experiments involving human serum were approved by the ethics committees of the Faculty of Tropical Medicine, Mahidol University (Submission number TMEC 12–014); of Sappasithiprasong Hospital, Ubon Ratchathani (reference 018/2555); and the Oxford Tropical Research Ethics Committee (reference 64–11). The study was conducted according to the principles of the Declaration of Helsinki (2008) and the International Conference on Harmonization Good Clinical Practice guidelines. Written informed consent was obtained from all patients enrolled in the study.

### Bacterial strains and plasmids

All bacterial strains and plasmids used are listed in **Table S1**. *Escherichia coli* strains DH5α and expression strains BL21(DE3)pLysS as well as the *B. pseudomallei* strain K96243 were cultured in lysogeny broth (LB) medium, LB agar or M9 minimal medium at 37°C. The concentrations of antibiotics added were as follows, unless stated otherwise: 100 μg/ml ampicillin (Ap, Sigma-Aldrich, Germany), 50 µg/ml kanamycin (Km, Carl Roth, Germany) and 25 μg/ml chloramphenicol (Cm, Sigma-Aldrich, Germany).

### Growth under stress conditions and isolation of intra- and extracellular proteins of *B. pseudomallei*

*B. pseudomallei* strain K96243 was cultured under the different conditions listed in **Table 1**. The cultures in experiments under stress conditions were generally grown to an optical density (OD 650 nm) of 0.5 and the different stressors were added for the time periods indicated. The selected different time periods for the different stress conditions (**Table 1**) were determined in preliminary experiments (unpublished data) to ensure a significant shift in expression pattern and sufficient starting material for subsequent analysis. By contrast, two different media (LB and M9 minimal media) were used in growth phase experiments over the total time period indicated. Afterwards, cells were harvested by centrifugation at 9,000 × g at 4°C for 5 min. Cell pellets were used immediately or stored at -20°C until use. The respective supernatants were centrifuged again at 10,000 × g at 4°C for 10 min to remove any remaining cells. Extracellular proteins in the supernatants were precipitated on ice using 10% (vol/vol) trichloroacetic acid for 24 h. After centrifugation at 15,000 × g at 4°C for 1 h, the supernatant was removed and the remaining protein pellets washed eight times with 100% ethanol, air-dried and, finally, dissolved in 8M/2M urea/thiourea and stored at -20°C until use. Regarding the isolation of intracellular proteins, the respective cell pellets were washed twice with ice-cold 10 mM Tris-EDTA pH 8.0 and resuspended in 1 ml of the same buffer. The resuspended cells were disrupted by a Ribolyser (Thermo Electron Corporation). Briefly, the cell suspensions were transferred to screw-cap microtubes (Sarstedt, Germany) containing 500 µl of glass beads (diameter, 0.10 to 0.11 mm; Sartorius, Goettingen, Germany). Cells were disrupted by homogenization using a Ribolyser at 6.5 m/s for 30 s. The lysate was centrifuged for 10 min at 21,000 × g (4°C). The centrifugation step was repeated at 21,000 × g (4°C) for 30 min in order to remove membrane fragments and insoluble proteins. The protein concentration was determined using Rotiquant (Roth, Germany) and the protein solutions were sterile filtered and stored at -20°C. Two biological replicates were done.

**Table 1:** List of growth and stress conditions for harvesting extra- and intracellular protein extracts.

Condition	Medium^a^	Stressor	Final concentration	Time point of harvest after stress
1. Stationary phase	LB	–	–	24h
2. Stationary phase	M9	–	–	48h
3. Anaerobiosis	LB	-O_2_/+NO_3_	–	24h
4. Acid stress	LB	pH 4.0	–	24h*
5. Oxidative stress	LB	H_2_O_2_	3 mM	2h*
6. Oxidative stress	LB	NO.	500 µM	3h*
7. Iron starvation	LB	2,2’-Bipyridyl	500 µM	4h*
8. Osmotic stress	LB	NaCl	470 mM	5h*

a Lysogeny-broth – LB, M9 Minimal medium – M9.* duration of stress.

### Two-dimensional gel electrophoresis

Two-dimensional polyacrylamide gel electrophoresis was performed using the immobilized pH gradient technique described previously (37). In the first dimension, the protein samples (40 μg in total) were separated on 7 cm immobilized pH gradient strips (SERVA Electrophoresis GmbH, Germany) with a nonlinear pH range of 3 to 10. For the second dimension, 12.5% polyacrylamide mini gels were run at 240 V and 50 mA at 21°C for 90 min. Subsequently, gels were either used for Western Blot experiments or stained with colloidal Coomassie brilliant blue for protein identifications. Stained gels were scanned with a light scanner with an integrated transparency unit (Quatographic, Braunschweig, Germany), as described previously (38). Both biological replicates were used for this study.

### Blood sera

A pool of 65 sera from culture-confirmed melioidosis patients from Ubon Ratchathani, Thailand, drawn at week 0 post admission were chosen as melioidosis positive sera [mel (+)], as described previously (39). Pooled negative serum [mel (-)], drawn from 25 healthy blood donors in the non-endemic area of Greifswald (Germany), served as a control to rule out previous episodes of undiagnosed melioidosis. Pools were used to gather antibody response patterns from multiple individuals. Further details about the sera and an extensive characterization can be found in our previous study (13). All sera were stored at -80°C until use.

### Western blot experiments

After separation by 2D polyacrylamide gel electrophoresis, proteins were blotted onto a nitrocellulose membrane in a semidry transfer approach (Peqlab Biotechnology GmbH). Gels and membranes were sandwiched between buffer-wetted filter papers. The transfer was performed at 1 mA current per 1 cm^2^ for 90 min (2D gels). The transfer quality was controlled by staining membranes reversibly with Ponceau S solution. After destaining twice with distilled water and once with tris-buffered saline (TBS), membranes were incubated with blocking buffer (5% bovine serum albumin [BSA] in 0.05 × TBS-T). The blocking step was carried out for 1 h at room temperature (RT) with constant agitation. Afterwards, membranes were washed five times with 0.05% TBS-T buffer and incubated with either 1:20000 pooled mel(+) or mel(-) sera diluted in 5% BSA in TBS-T at 4°C with constant agitation overnight. Washing steps with 0.05 TBS-T were repeated five times and membranes were incubated with 1:50000 anti-human IgG antibodies (Anti-human IgG, Fc-specific, HRP, Jackson Immunoresearch, UK) diluted in 5% BSA in TBS-T on a tumble shaker for 1 h at RT. Membranes were washed again five times with 0.05% TBS-T and incubated with Luminol for 1 min in the dark at RT. Finally, signals were detected by a Fusion FX 7 Imager (Peqlab GmbH/VWR, Germany) after different time points (30, 60, 120, 180, 240 and 360 s) and recorded as graphic files (*.tif).

### Protein quantitation and mass spectrometry (MALDI-TOF) approaches

The 2D gel and Western blot images were analyzed with Delta2D software (Decodon GmbH, Greifswald, Germany), as described by Holtfreter and colleagues (40). A fused image of all 2D Western blots [two mel(+) and two mel (-)] per stress condition was obtained using the union fuse option of Delta2D. Spots on the fusion image were automatically detected and manually validated by comparing the original blot images with the fusion image. Subsequently, the spot map and the corresponding labels from the fusion image were transferred to all blot images obtained under a specific condition, ensuring a uniform analysis. The raw volume data were analyzed, due to a strong difference in signal intensity of the Western blots. Several Western blot spots of the same protein on one gel were averaged to one single signal value.

The fused 2D Western blot images were further matched with the colloidal Coomassie-stained gels to identify the proteins corresponding to the 2D Western blot spots. Afterwards, selected proteins were excised from colloidal Coomassie-stained gels and peptides were prepared for MALDI-TOF‐MS by trypsin digestion. Therefore, the gel pieces were washed twice with 100 µL of a solution of 50% CH_3_OH and 50% 50 mM NH_4_HCO_3_ for 30 min and once with 100 µL 75% CH_3_CN for 10 min. After drying at 37°C for 17 min, 10 µL of a 4 µg/mL trypsin (Promega, Madison, WI, USA) solution were added and the mixture was incubated at 37°C for 120 min. Subsequently, gel pieces were covered with 60 µL 0.1% trifluoroacetic acid in 50% CH_3_CN for extraction and incubated for 30 min. The peptide containing supernatant was transferred to a new microtiter plate and the extraction was repeated with 40 µL of the same solution. The supernatants were completely dried at 40°C for 220 min. The dry peptides were resuspended in 0.9 µL of α-cyano-4-hydroxycinnamic acid matric (3.3 mg/mg/ml in 50/49.5/0.5% (v/v/v) CH3CN/H2O/trifluoroacetic acid), respectively, and 0.7 µL of the solution obtained was deposited on the MALDI target plate. The samples were dried on the target plate for 10 to 15 min before measurement in the MALDI-TOF instrument. The MALDI-TOF measurement was carried out on the AB SCIEX TOF/TOFTM 5800 Analyzer (AB Sciex/MDS Analytical Technologies). The spectra were recorded in a mass range from 900 to 3700 Da with a focus mass of 1700 Da. To obtain a single spectrum, 25 sub-spectra with 100 shots per sub-spectrum were accumulated using a random search pattern. If the autolytical fragment of trypsin with the monoisotopic (M+H)+ m/z at 2211.104 reached a signal to noise ratio of at least 40, an internal calibration was automatically performed as one-point-calibration using this peak. The standard mass deviation was less the 0.15 Da. If the automatic mode failed (in less than 1%), the calibration was carried out manually. The five most intense peaks from the time of flight spectra were selected for MS/MS analysis. A single spectrum was obtained by the accumulation of 20 sub-spectra with 125 shots per sub-spectrum using a random search pattern. The internal calibration was automatically performed as one point calibration with the monoisotopic arginine (M+H)+ m/z at 175.119 or Lysine (M+H)+ m/z at 147.107 reached a signal to noise ratio of at least 5. The peak lists were created by using GPS ExplorerTM Software Version 3.6 (build 332) with the following settings for TOF-MS: mass range, 900–3700 Da; peak density, 20 peaks per 200 Da; minimum signal to noise ratio of 10. All peak lists were analyzed using the Mascot search engine version 2.4.0 (Matrix Science Ltd., London, UK) with a specific *B. pseudomallei* strain K96243 sequence database. Some spots lead to double, triple or quadruple identifications, in extra- as well as intracellular gels. These identifications were included in the study and regarded as single candidates.

### *B. pseudomallei* antigen selection, cloning, expression and purification

*B. pseudomallei* proteins with highly increased signal intensities in mel (+) sera were chosen as targets for dot blot analyses (**Table S2**). In addition, we complemented our pool of experimentally identified serodiagnostic antigen candidates with a set of further *B. pseudomallei* proteins (**Table S3**): protein antigens that have been used as serodiagnostic biomarkers in a protein microarray (28, 41, 42), peptide array (14), ELISA (15, 43, 44) or lateral flow assay (16) served as controls for our test setup. In addition, we included an antigen that has been successfully used in an ELISpot assay (27), proteins that contribute to *B. pseudomallei* virulence (45–49) and vaccine candidates (48, 50–55). As the latter should ideally induce a strong immune response, these proteins might also be valuable serodiagnostic antigens, which, indeed, has been shown, for example, for Hcp1 (16). Seven further undescribed proteins were available in our laboratory and included in this study. The green fluorescent protein serves as a negative control. Proteins were subjected to an analysis regarding their potential diagnostic performance, as described by Felgner et al. (28), including their genomic location (Chromosome 1 or 2), bacterial compartment (cytoplasm, extracellular, periplasm, membrane/outer membrane), predicted function (e.g. protein folding and stabilization, metabolism, virulence, unknown function) and, first of all, solubility in phosphate-buffered saline after the freezing and storage process. Finally, all protein antigens were analyzed by PSORTb version 3.0.2 (http://www.psort.org/psortb/), and any signal sequences or transmembrane domains (predicted by the TMHMM Server v. 2.0; http://www.cbs.dtu.dk/services/TMHMM/) were excluded for further cloning. The respective protein-encoding DNA fragments were amplified by polymerase chain reaction using specific oligonucleotides (**Table S4**) and genomic DNA from *B. pseudomallei* K96243 strain as the template. The polymerase chain reaction products were digested and cloned using appropriate restriction enzymes and protein expression plasmids (**Table S4**). The DNA sequence of all cloned genes was confirmed by Sanger sequencing. Regarding protein expression, plasmids were transformed in *E. coli* expression strain BL21(DE3)pLysS by heat shock and grown in LB medium with permanent agitation at 37°C to an optical density (OD540 nm) of 0.5. Protein expression was induced by adding isopropyl-β-D-thiogalactopyranoside (1 mM final concentration) (Carl Roth GmbH, Germany), for the time and temperature indicated (see **Table S4**), cells were harvested by centrifugation at 8,000 × g at 4°C for 10 min. Subsequently, cells were disrupted by six cycles (3 min at 4°C) of ultrasonic homogenizer UP50H (Hielscher Ultrasonics GmbH, Germany), and the lysates were centrifuged 12,000 × g at 4°C for 30 min. Supernatants were stored at -20°C until use. The protein purification of Strep- or His-tag recombinant proteins was performed by using Gravity flow Strep Tactin-Sepharose Columns (IBA GmbH, Göttingen, Germany) or Ni-NTH Agarose (Qiagen, Germany), according to the manufacturers’ instructions. If solubility enhancement tags were used for protein expression, they were removed by His-tagged Tobacco Etch Virus protease cleavage over night at 4°C. Solubility enhancement tags and His-tagged Tobacco Etch Virus were separated from the protein of interest by a second Ni-NTH Agarose step, whereas the protein of interest eluted in the flow through. Afterwards, purified proteins were dialyzed against Dulbecco’s phosphate-buffered saline (Gibco-life technologies, USA) or were dissolved in 8 M Urea, and their purity was confirmed by SDS-PAGE. Recombinant proteins were stored at -20°C until use for dot blot analyses.

### Dot blot analyses

Concentrations of all purified recombinant proteins were measured using Roti^®^-Quant (Carl Roth, Germany), according to the manufacturer’s instructions. Afterwards, dot blot analysis was performed using the Bio-Dot^®^ Microfiltration apparatus (Biorad, Germany). For that, nitrocellulose membranes (0.45 µm, GE healthcare, USA) were used and 20 µl protein solution with a concentration of 0.05 µg/µl was applied per dot (in total 1 µg/protein). For this purpose, we used two biological replicates and calculate four ratios per time point in total. All four ratios had to be higher than 3 in case of a melioidosis biomarker and were averaged to the mean ratio per protein. As discussed below (see results), additional dot blot experiments were carried out at 0.1, 0.2, 0.3 or 0.5 µg protein per spot for selected proteins using three biological replicates and calculated three ratios per time point in total. The subsequent procedure is based on the Western blots described above. Briefly, membranes were blocked with blocking buffer (5% BSA in 0.05 × TBS-T). Membranes were washed and then incubated with 1:20000 pooled human mel (+) or mel (-) sera diluted in TBS-T supplemented with 5% BSA at 4°C overnight. After washing steps with 0.05× TBS-T, the membranes were incubated with 1:50000 anti-human IgG antibodies (Anti-human IgG, Fc-specific, HRP, Jackson Immunoresearch, UK) diluted in 0.05 TBS-T supplemented with 5% BSA at RT for 1 h. Membranes were washed with 0.05 × TBS-T and incubated with Luminol at RT for 2 min in the dark. Finally, signals were detected by Fusion FX 7 Imager (Peqlab GmbH VWR, Germany) after different time points (180 and 360 s) and recorded as graphic files (*.tif). The images were analyzed using Delta2D (Decodon GmbH, Greifswald, Germany).

### Statistical analyses and software used

Delta2D was used for the analyses of the 2D Western blot experiments. The mean spot intensity of both positive and negative replicates were determined for each spot. The ratio of these mean spot intensities (mel (+) vs. mel (-) sera) was calculated to quantify the serodiagnostic potential of immunoreactive proteins. Spots with at least a threefold signal increase in mel (+) blots compared to mel (-) blots were classified as melioidosis biomarkers candidates. Delta2D was also used for the analyses of the dot blot experiments. In case of dot blot experiments using 1µg/dot, two replicates were analyzed. The respective spot intensities (raw signals) of the mel(+) and mel(-) dot blots were computed, ultimately yielding four ratios: R1=mel(+)1/mel(-)1; R2=mel(+)2/mel(-)2; R3=mel(+)1/mel(-)2; and R4=mel(+)2/mel(-)1). All ratios had to be greater than 3 for a protein to be considered a potential candidate for further analysis (dot blot analyses with protein amounts of 0.1 to 0.5 µg/dot). In case of dot blots using 0.1 to 0.5 µg/dot of applicated proteins, we analyzed triplicates. The respective spot intensities (raw signals) of the mel(+) and mel(-) dot blots were computed, ultimately yielding three ratios: R1=mel(+)1/mel(-)1; R2=mel(+)2/mel(-)2; R3=mel(+)3/mel(-)3. Here, the averaged ratios again had to be greater than 3 for a protein to be considered a potential candidate for further studies and *t-*test analyses were performed. Hierarchical clustering of IB detection ratios were generated with the Multi Experiment Viewer 4.9.0 (MeV 4.9.0, USA) using following parameters: Linkage method: complete linkage; Distance metric selection: pearson correlation. Excel 2016 (Microsoft Corporation, USA), GraphPadPrism 6.0 (GraphPad software, Inc., USA.) and Multi Experiment Viewer 4.9.0 (MeV 4.9.0, USA) were used for further data analyses and data visualization.

## Results

### Design of a comprehensive immunoproteome approach

**Figure 1:**
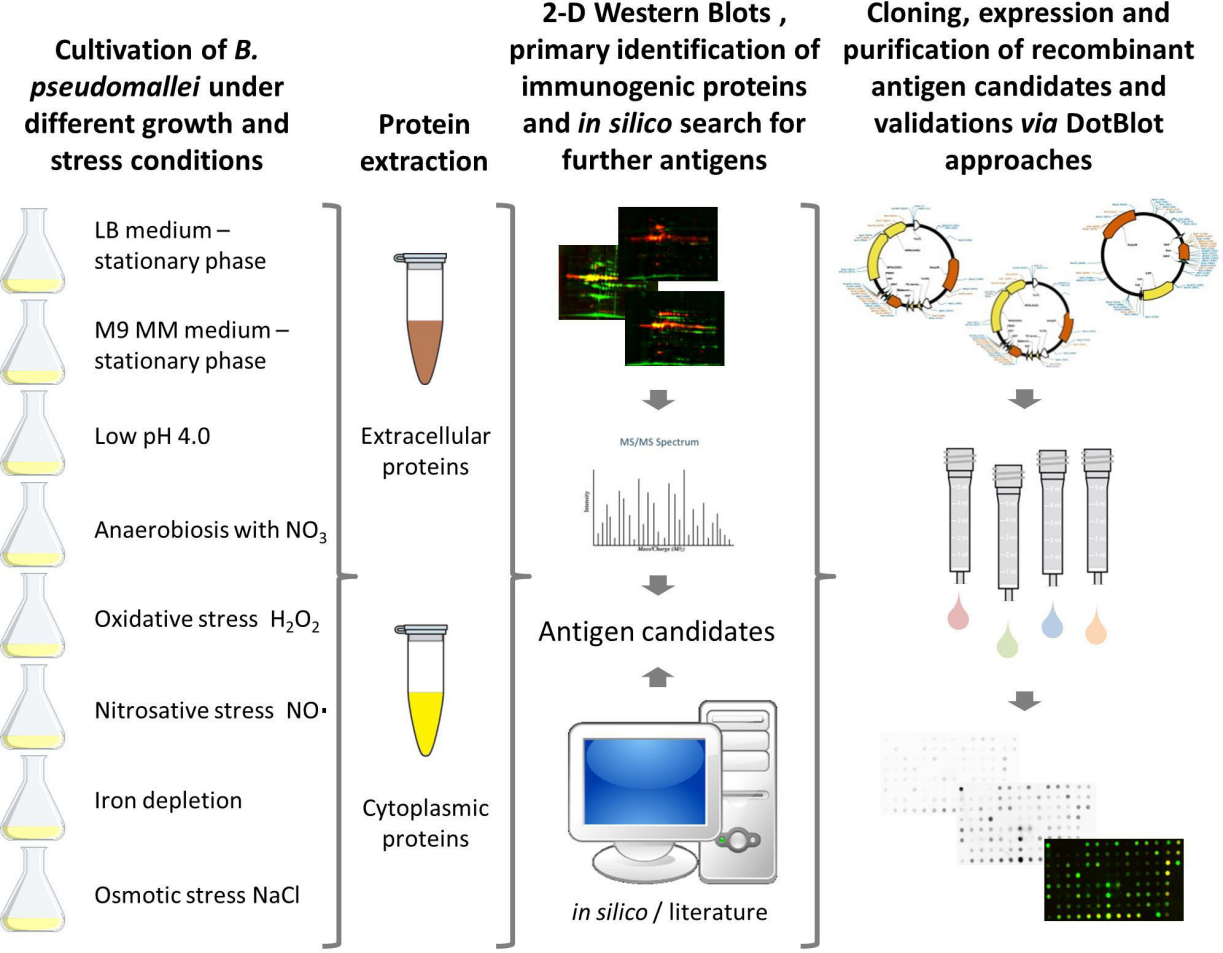
Workflow for the detection of serodiagnostic biomarker proteins of *Burkholderia pseudomallei* using a comprehensive immunoproteomic approach. *B*. *pseudomallei* K9 was cultured under different growth and stress conditions to increase the total number of proteins expressed. Subsequently, cytoplasmic and extracellular protein fractions were extracted and candidate biomarkers were identified by two-dimensional (2D) Western blots by incubation with melioidosis-positive sera or negative controls. Immunogenic proteins identified and literature targets were amplified by polymerase chain reaction, cloned into different expression vectors and expressed in *E*. *coli*. Finally, dot blot analyses of the recombinant proteins were used as a confirmatory test for the 2D Western blot analyses.

A large proportion of the genes in *B. pseudomallei* display a condition-dependent and unpredictable expression (56). Conventional approaches to biomarker identification, usually based on a single growth condition, might, therefore, be biased because many proteins are not expressed at all. In order to address this issue, we chose eight different growth and stress conditions in the study presented here to increase the number of expressed, hence identifiable, protein antigens (**Figure 1**). We focused on stress conditions that mimic conditions encountered during an infection in the human host: pH stress (1), osmotic stress (2), iron depletion (3), anaerobic growth condition (4), oxidative stress by reactive oxygen species (ROS) (5) and reactive nitrogen species (RNS) (6), respectively. In addition, we included bacterial growth to stationary phase in two different media [LB broth (7) and M9 minimal medium (8)] (**Table 1**; **Figure 1**). The biomarker candidates were identified based on an immunoproteomics approach analyzing intra- and extracellular protein extracts obtained from *B. pseudomallei* grown under the aforementioned stress conditions.

### A diverse set of stressors aiming to mimic host-like habitats induce a pronounced proteome shift and enable the identification of *B. pseudomallei*-specific biomarkers

**Figure 2:**
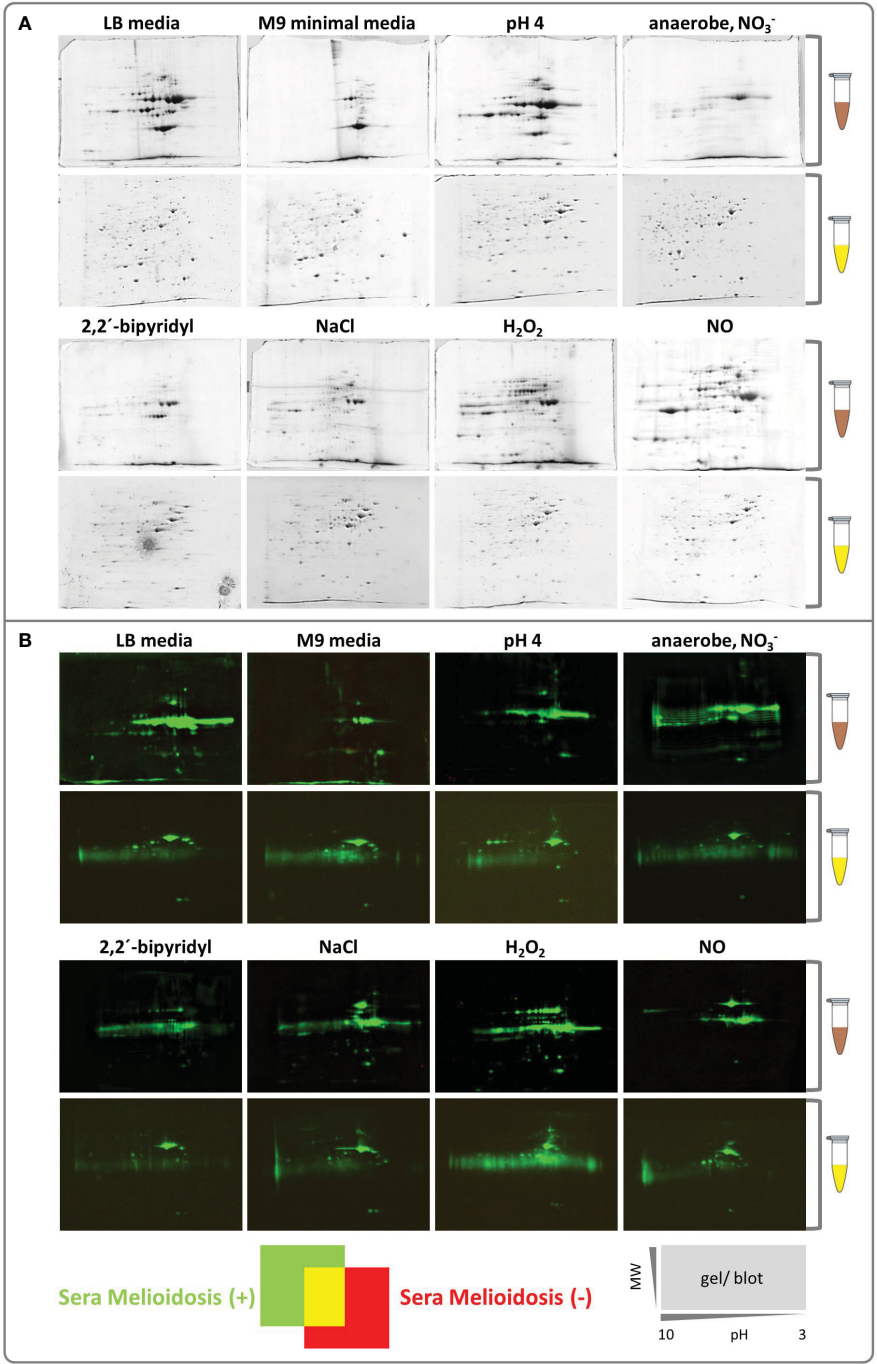
Colloidal Coomassie stained 2D gels and Western blots of intracellular and extracellular *B*. *pseudomallei* protein fractions. **(A)** Extracellular (brown tube) and intracellular proteins (yellow tube) were separated by 2D gel electrophoresis using immobilized pH gradient strips, the resulting gels were stained with Silver Blue colloidal Coomassie (CBB G-250) and used for protein identification by MALDI-TOF MS. Gels are shown from pH 10 to 3 (left to right side/gel). **(B)** Dual channel images of 2D gel Western blots generated with the Delta2D software (Decodon GmbH), showing the differences in the protein patterns of Western blots detected incubated with sera from melioidosis patients (green) or healthy blood donors (red). A gel/Western blot scheme shows the decreasing molecular weight (MW, on the y-axis) and the isoelectric point (pH 10 to 3, on the x-axis).

After exposing *B. pseudomallei* cells to different stressors (**Table 1**), the extra- and intracellular proteins were extracted separately. In contrast to the intracellular proteome of *B. pseudomallei* (52, 53), the effect of different stressors on the extracellular proteome (not only the immunoproteome) has not been studied extensively. Therefore, we also performed an extensive analysis of the extracellular protein fraction, which, indeed, showed a strong shift depending on the stressor/growth condition, consistent with our hypothesis (**Figure 2A**). Identifying all visible protein spots by mass spectrometry, we found the following numbers of proteins under the respective condition: RNS stress (80), osmotic stress (50), ROS stress (34), acid stress (27) and stationary phase of LB medium (26). Far fewer proteins were identified under iron-deficiency stress (13), growth in M9 minimal medium (7) and anaerobic conditions (5) (**Table S5**). Therefore, as expected, additional growth conditions increase the number of proteins expressed (**Figure 2**) and, thus, the chance to identify novel, highly immunogenic protein biomarkers

**Figure 3:**
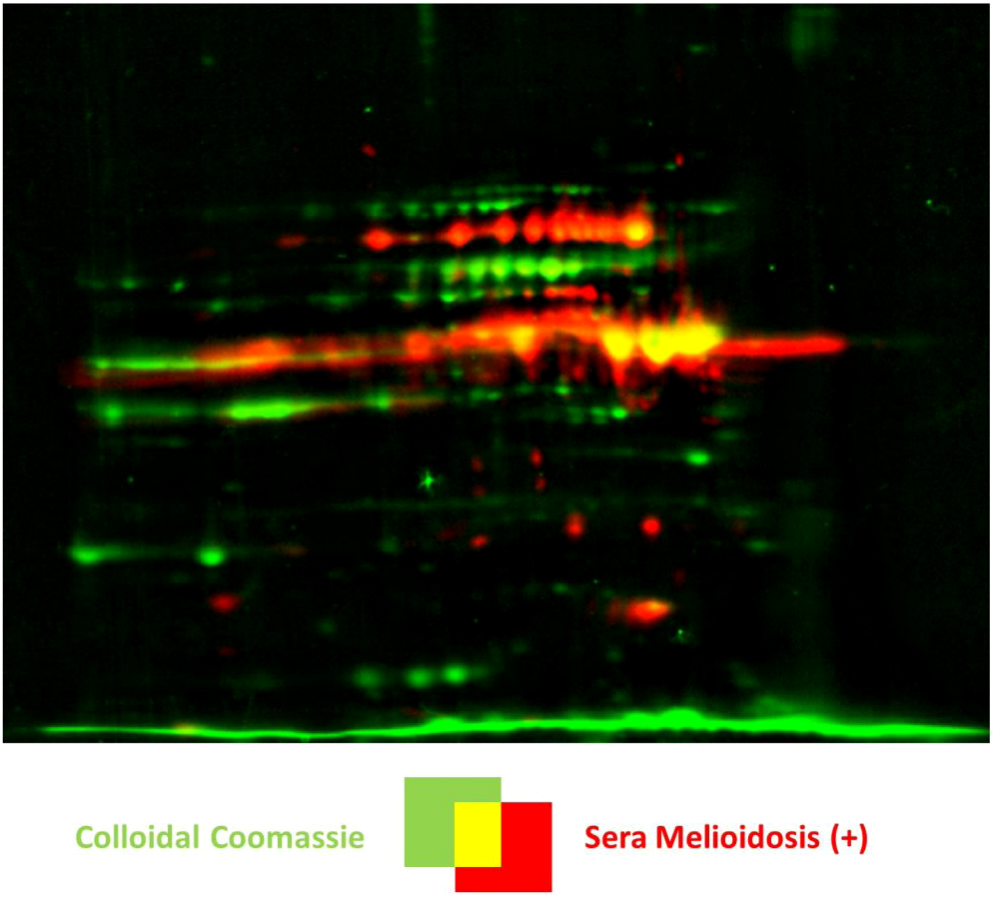
Identification of melioidosis biomarkers by a dual-channel image representation of a 2D gel and the respective Western blot by the Delta2D software. A representative image is shown, in this case, of the extracellular protein fraction of *B*. *pseudomallei* K9 cultured under oxidative stress with 3 mM H_2_O_2_. The colloidal Coomassie stained gel is shown in green and the Western blot in red. Positive candidates are shown in yellow, and proteins not recognized by the melioidosis-positive sera are shown in green. Note that signals that do not correspond to proteins on the Coomassie stained gels are shown in red.

**Figure 4:**
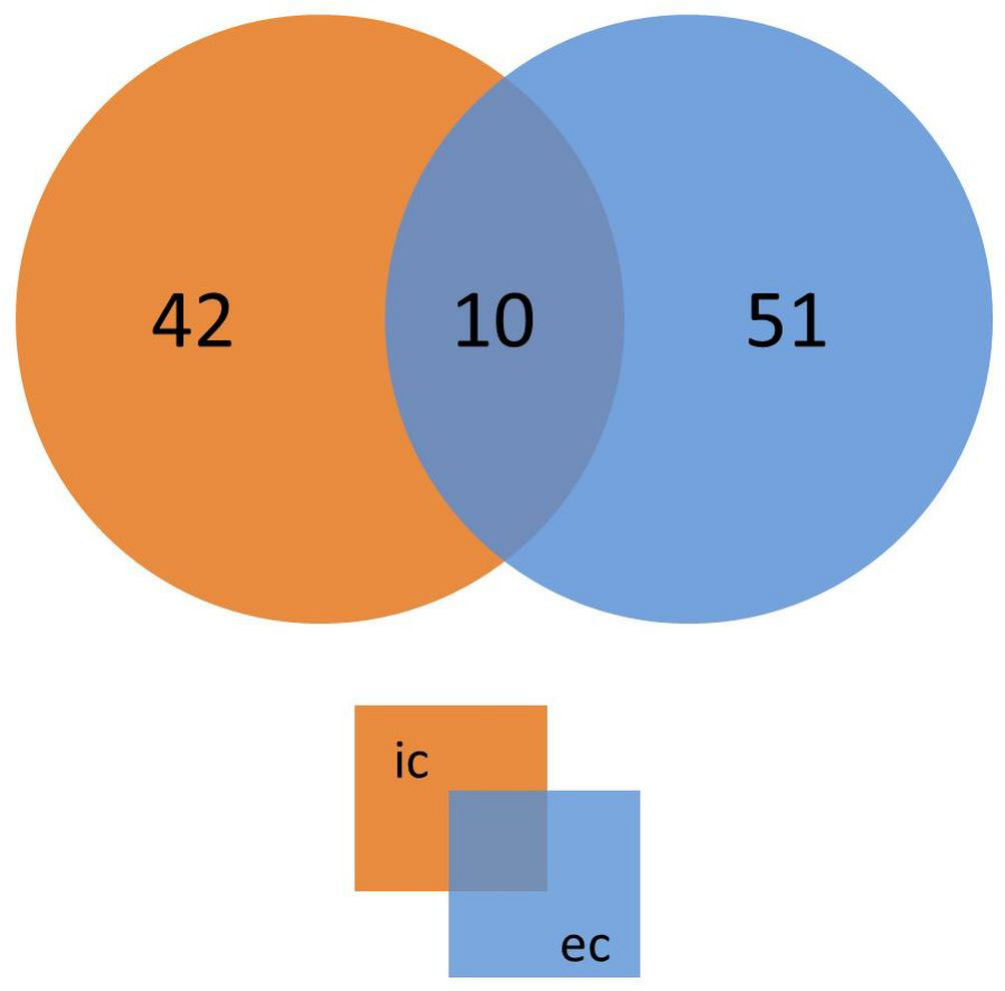
Number of putative immunogenic proteins identified from extracellular and intracellular protein fractions. The Venn diagram shows the number of proteins with specific IgG signals obtained in immunoblot experiments using extracellular (ec, blue) or intracellular proteins (ic, orange). The overlap represents proteins identified in both fractions.

We next identified the patient IgG reactive protein spots by IB experiments probing intra- and extracellular protein fractions, respectively. Indeed, we found many strong IgG signals on IB incubated with mel (+) sera (**Figure 2B**) with no detectable background from the controls. These IgG reactive biomarkers were subsequently identified from preparative gels by MALDI-TOF MS/MS. An overlay of the images of the colloidal Coomassie-stained gels and the IBs served as matrices for protein identification (**Figure 3**). Proteins that showed at least threefold signal induction in patient sera compared to the controls were defined as seroreactive antigens (**Table S6**). We identified a total of 103 different immunogenic proteins, 61 seroreactive proteins resulting from the extracellular protein fractions and 52 from the intracellular protein fraction. Only 10 proteins were found in both protein fractions (**Figure 4**).

**Figure 5:**
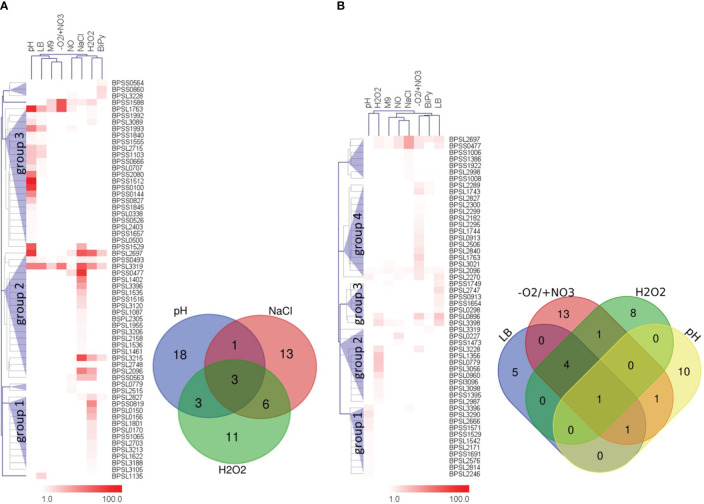
Hierarchical clustering of IB detection ratios and Venn diagrams of the respective proteins expressed under the respective stress conditions. The hierarchical clustering trees were based on the IgG signal ratios of the IB experiments obtained using the extra- **(A)** or intracellular **(B)** protein fraction. The protein clusters are shown as blue triangles and are indicated as respective groups. Extracellular groups: 1 – mainly ROS stress, 2 – mainly osmotic stress, 3 – mainly acid stress. Intracellular groups: 1 – mainly acid stress, 2 – mainly ROS stress, 3 – mainly growth stationary phase LB medium and 4 – mainly anaerobic growth conditions. The corresponding Venn diagrams show the distributions of the proteins of these stress and/or growth conditions singled out for biomarker identification. M9 – stationary phase in M9 minimal medium; LB – stationary phase in LB medium; pH – acidic stress pH 4.0; -O2/+NO3 – anaerobic growth with nitrate; BiPy – iron deficiency by 2,2′-Dipyridyl; NaCl – osmotic stress caused by sodium chloride; NO – RNS stress caused by MAHMA NONOate; H2O2 – ROS stress due to hydrogen peroxide.

The hierarchical clustering of IB results of the extracellular protein fractions revealed three big protein clusters consisting of candidates which were mainly detected after acid stress, followed by osmotic and oxidative stress (**Figure 5A**). The three stressors provoked the expression of about 90% (55/61) of all immunogenic extracellular proteins identified. Moreover, there was only a little overlap between them (**Figure 5A**, Venn diagram). Only three immunogenic proteins were detected in all extracellular fractions under these three conditions, 10 proteins in at least two different protein fractions, but 42 proteins exclusively in only one of the three protein fractions. Six proteins were solely expressed in two or one of the remaining stress conditions: RNS and iron deficiency (one protein), RNS stress (two proteins), iron deficiency (two proteins) and stationary phase in LB medium (one protein) (**Figure 5**; **Table S6**). Neither growth in M9 minimal medium nor anaerobic growth conditions lead to the identification of any additional proteins in the IB experiments of the extracellular fractions.

The same analyses using the intracellular fractions revealed four large protein clusters (**Figure 5B**). Overall, one protein (BPSL2096) showed increased ratios under all eight conditions, one protein (BPSL2697) was found under seven conditions (all except pH stress) and another one (BPSS0477) was detected under six different stress conditions (all except pH and iron deficiency). However, most immunogenic candidates were found after the hypoxic growth condition, followed by acid stress, ROS stress and growth to stationary phase in LB medium (**Figure 5B**; **Table S6**). A total of 85% (44/52 proteins) of all immunogenic intracellular proteins identified belong to these fractions. Thirty-six of these proteins were exclusively detected in one of the intracellular fractions (stationary phase LB medium: 5 proteins, anaerobic growth: 13 proteins, ROS stress: 8 proteins and acid stress: 10 proteins) (**Figure 5B**, Venn diagram). Only one protein was found in all four protein extracts, five proteins in at least three extracts and two proteins in at least two protein extracts (**Figure 5B**, Venn diagram). The remaining eight proteins were detected in protein fractions obtained after osmotic stress (five proteins), RNS stress (two proteins) and iron deficiency (one protein) (**Figure 5B**; **Table S6**). As observed for the extracellular proteins, M9 medium did not lead to the identification of any additional immunogenic protein in the intracellular protein fraction.

Combining the results of extra- and intracellular fractions, most candidates were found under the following conditions: acid stress (37 proteins), ROS stress (35 proteins), osmotic stress (28 proteins), late stationary growth phase in LB medium (25 proteins) and after growth under hypoxic growth conditions (24 proteins). These five cultivation conditions gained 95% (98/103) of all immunogenic proteins found in the 2D IB experiments of this study.

### Functional categorization of the immunogenic proteins identified

**Figure 6:**
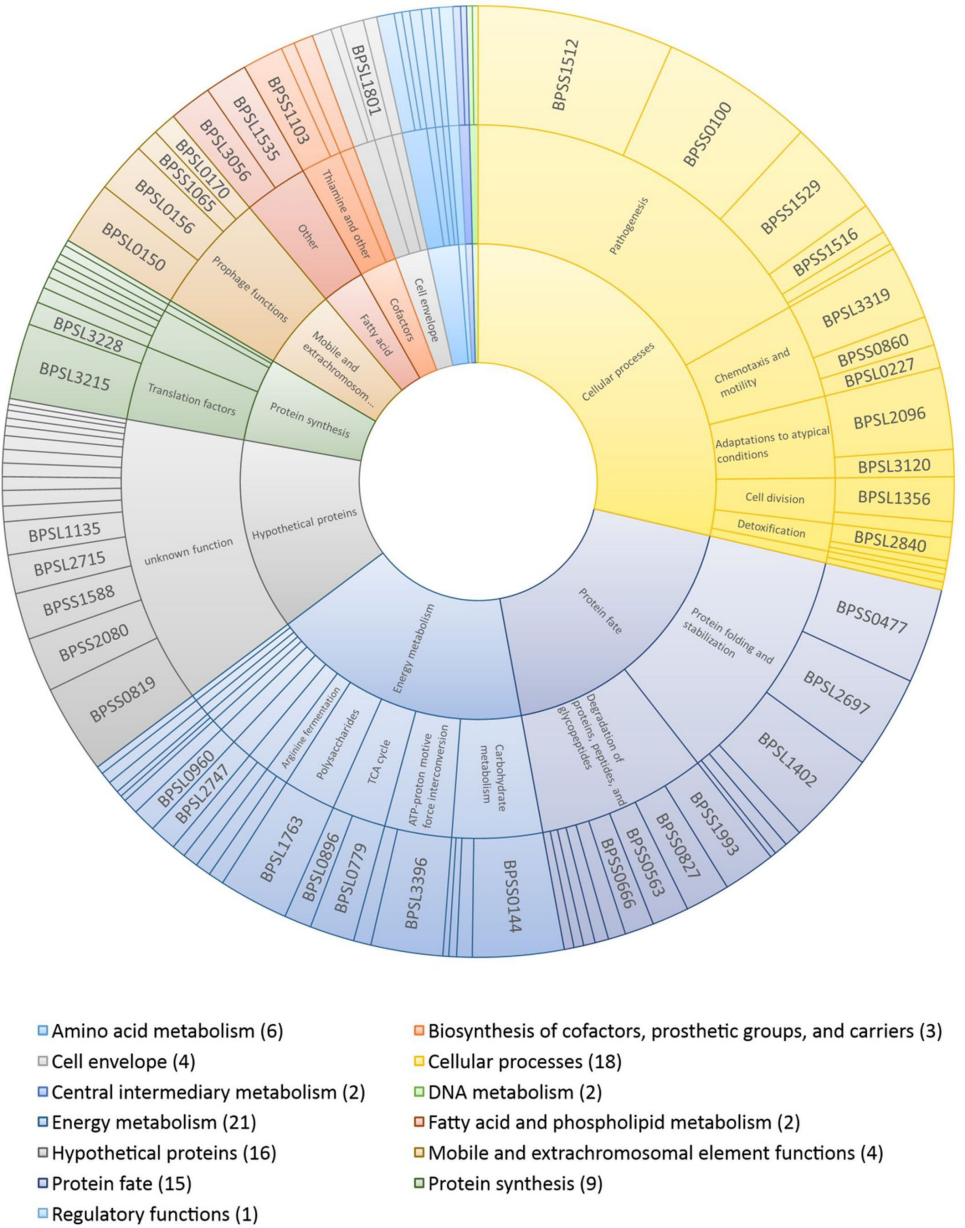
Sunburst chart showing the induction ratios of the putative immunogenic proteins identified and their functional classifications. The outer ring indicates individual proteins and their sizes indicate their average induction ratios measured in melioidosis-positive sera compared to control sera. The inner ring represents the main role, and the middle ring the sub role. The functional categorizations (main role, sub role) were taken from the Kyoto Encyclopedia of Genes and Genomes database (https://www.genome.jp/kegg/). The number of proteins identified is given in brackets after each group.

The immunogenic proteins identified were classified according to their specific cellular roles (**Table S6**; **Figure 6**). Most proteins belong to energy metabolism (21), cellular processes – including pathogenesis, adaptation to atypical conditions and detoxification – (18), protein fate and/or modification (15) and protein biosynthesis (9). Additionally, 24 proteins are involved in the amino acid metabolism (6), extra chromosomal element functions (4), cell envelope (4), biosynthesis of cofactors and prosthetic groups (3), DNA metabolism (2), central intermediary metabolism (2), fatty acid and phospholipid metabolism (2), and regulatory functions (1). The remaining 16 proteins were classified as hypothetical proteins with unknown functions.

High ratios of immunoblot signals of melioidosis to control sera were measured for candidates already described in the literature (**Table S6**) and new candidates, for example, for molecular chaperones (BPSL2697 – GroEL, BPSS0477 – GroEL2) (13, 19, 28), a chitin degradation enzyme (BPSL1763), flagellin (BPSL3319) (41) and the alkyl hydroperoxide reductase subunit C (BPSL2096) (13, 19, 41). These proteins are important for the maintenance of the bacterial physiology, and, hence, were found in at least seven different intra- and/or extracellular protein fractions. However, the proteins giving the highest IgG signals, including TssM (BPSS1512) (28), VasD (BPSS0100) and BipD (BPSS1529) (57), are involved in the pathogenesis of *B. pseudomallei*, but were detected only in the extracellular protein fraction after acid or osmotic stress, respectively. Other proteins leading to high IgG signals, such as BPSL1402 (trigger factor), BPSS0144 (putative amylase) and BPSS0819 (hypothetical protein), were also only detected under a single condition and are involved in protein biosynthesis, carbohydrate metabolism or have an unknown function.

### Selection and validation of seroreactive protein antigens by dot blot experiments

The limiting factor for the protein identification by mass spec in IB experiments is the resolution of the 2D gels, as overlapping and unresolved protein spots might lead to false results. Therefore, biomarker candidates were heterologously expressed and their serodiagnostic potential was subsequently evaluated by dot blot analysis in the case of successful, soluble protein expression.

**Figure 7:**
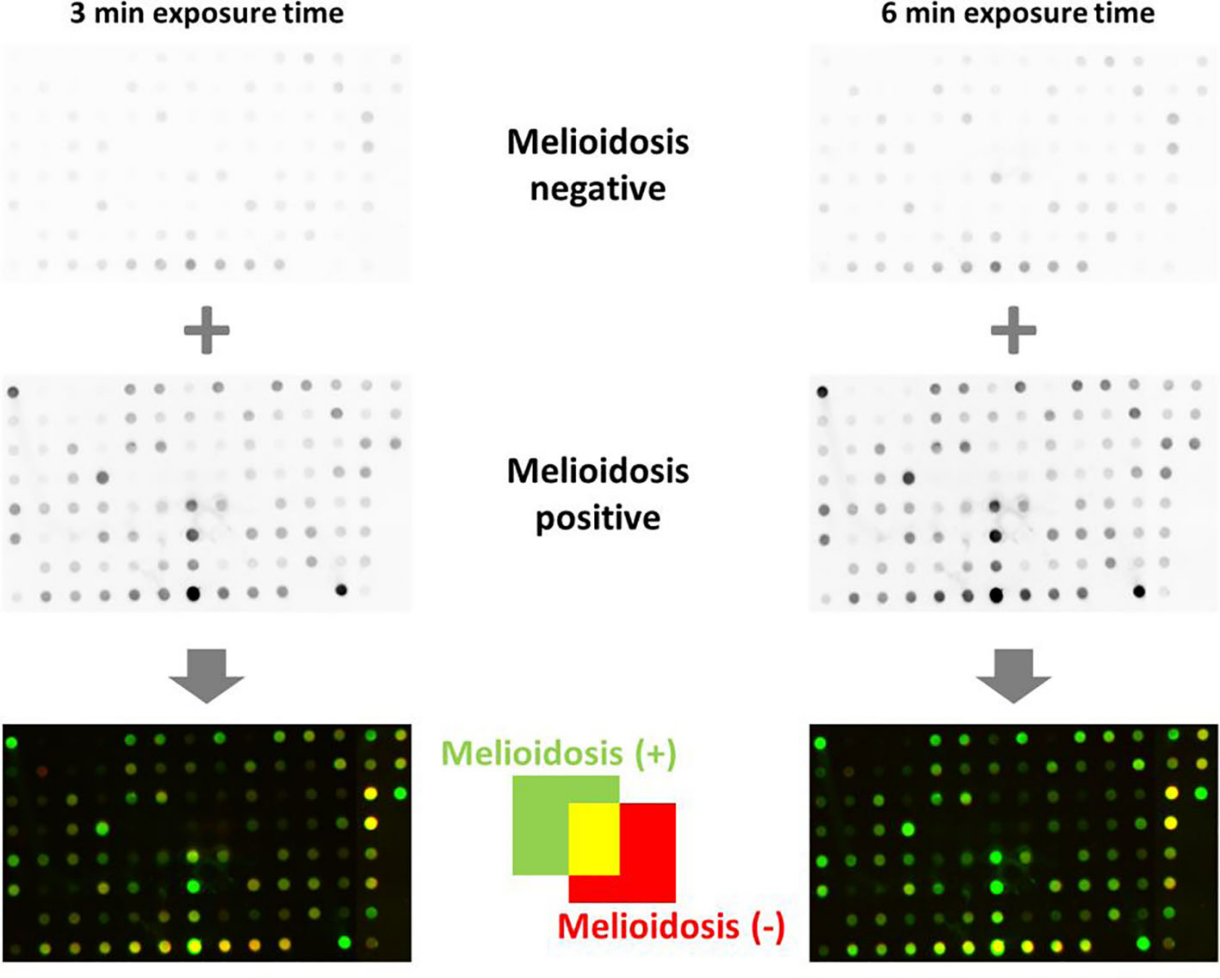
Dot blot analyses of *B. pseudomallei* recombinant protein biomarker candidates. A volume of 1 µg per protein was blotted onto nitrocellulose membranes and incubated with melioidosis-positive sera and negative controls. Proteins shown as green dots in the merged blots (bottom) are recognized only by melioidosis-positive sera, red dots are recognized only by sera of healthy blood donors and yellow dots are proteins recognized by both classes of sera. One of two technical replicates is shown.

From the 103 potential antigens identified, 63 proteins were successfully purified (**Table S2**) using different plasmid and expression strategies (see Material and Methods). Among them, at least 17 identified proteins (**Table S6**) were already described, corroborating the validity of our approach. In addition to our experimentally identified antigens, we also expressed and purified literature targets, for example, serodiagnostic marker and vaccine candidates (provoking a strong immune response), see Methods and **Table S3**. Thus, we analyzed a total of 101 different *B. pseudomallei* proteins in our Dot Blot experiments using purified recombinant proteins on nitrocellulose membranes to examine any antibody reactivity (**Figure 7**).

**Figure 8:**
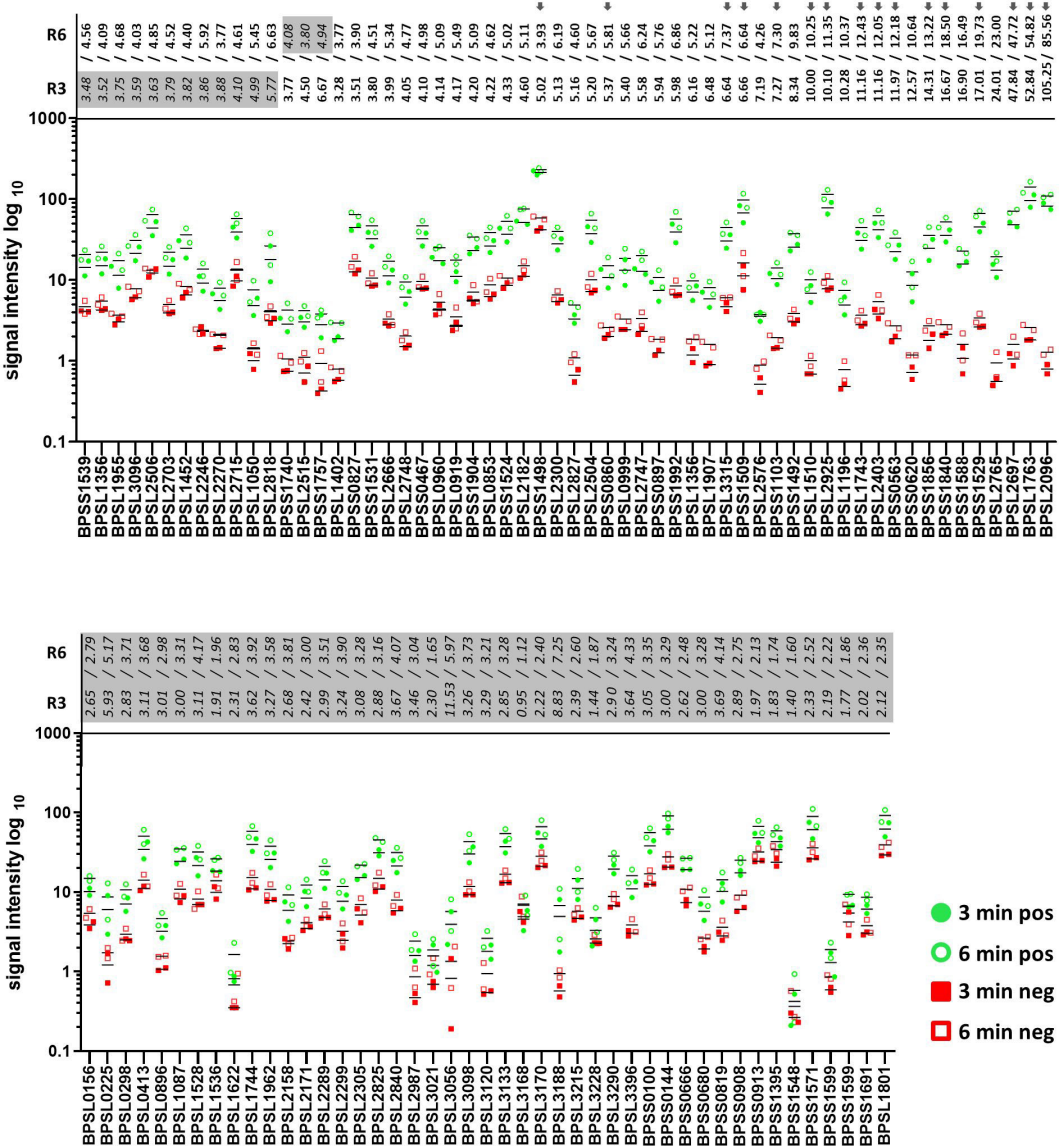
Signal intensities and the calculated ratios of all recombinant proteins of *B*. *pseudomallei* measured by dot blot analysis. Signal intensities of the replicates incubated with melioidosis-positive sera (green circle) and sera of healthy blood donors (red square) are shown. Blots were exposed for 3 (filled circle and square) and 6 min (no filling). Averaged ratios were calculated using all possible combinations of the signal intensities measured (a total of four ratios/exposure time of 3 or 6 min) and are written at the top of both graphs (3 min – R3 and 6 min – R6). Only recombinant proteins with all four ratios above 3 were considered as significant candidates (bold, top graph); all other candidates are considered as nonsignificant (italic and highlighted in gray, top and bottom graph). Grey arrows indicate proteins subjected to further dot blot analyses using different protein concentrations (**Figure 9**).

Signal intensities of single dots enable the calculation of the respective intensity ratios for mel (+) to mel (-) sera. Fifty-eight proteins showed induction ratios of 3 and higher, whereas 45 recombinant proteins did not fulfill these criteria (**Figure 8**). Of the 58 proteins that fulfilled our criteria, 15 gave induction ratios above 10, another 15 showed ratios higher than the well-known antigen BPSS1498 and the remaining 28 proteins showed ratios between 3.28 and 9.83 (**Figure 8**).The highest induction ratios were observed for BPLS2096 (above 105), BPSL1763 (above 50), BPSL2697 (about 49), BPSL2765 (about 24), BPSS1529 (about 18), BPSS1588 (about 16), BPSS1840 (about 16), BPSS1856 (about 14), BPSS0620 (about 12), BPSS0563 (about 12), BPSL2403 (about 11), BPSL1743 (about 11), BPSL1196 (about 10), BPSL2925 (about 10) and BPSL1510 (about 10) (**Figure 8**). It is noteworthy that, in agreement with the literature (15, 16), the strongest emitter-coupled logic signals were measured for BPSS1498, which is a well-known serodiagnostic marker, hence, we attributed the background to the rather high protein concentration in the dot blot experiments in **Figure 8**.

Since it is probable that the optimal spotting concentration varies from antigen to antigen, we repeated the experiments using five additional dilutions (0.1 to 0.5 µg per dot) of a selected set of the most promising candidates (BPSL1763, BPSL2403, BPSL2925, BPSS1840, BPSL3315, BPSS1509, BPSL1510, BPSL1743, BPSS0563, BPSS1103, BPSS1856 and BPSS0860) and included the four well-described melioidosis biomarker proteins: BPSS1498, BPSS1529, BPSL2096 and BPSL2697 (13, 16, 19, 28).

**Figure 9:**
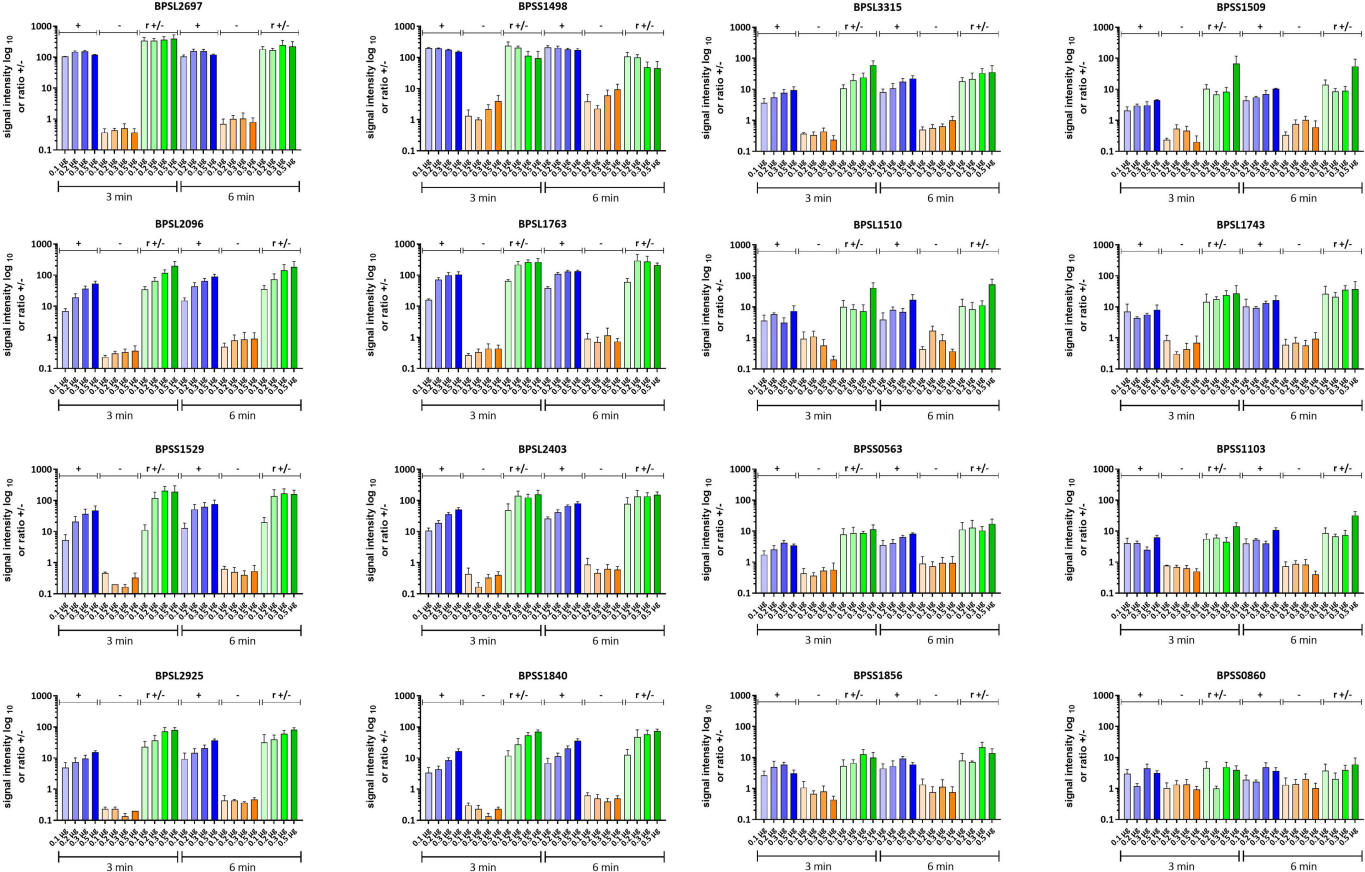
Dot blot analysis of selected recombinant *B*. *pseudomallei* proteins. Signal intensities of three technical replicates incubated with melioidosis-positive sera (blue, +) and sera from healthy blood donors (orange, -) are shown. The blots were exposed for 3 and 6 min, respectively, and the averaged ratios (r +/-) were calculated from the signal intensities of the biological replicates and are shown in green. Means of three independent experiments are shown. Error bars indicate the standard error of the mean. Increasing color intensities indicate an increase in the protein concentration per dot.

As expected, our experiments clearly show that the optimal spotting condition regarding signal intensity and background is different for the respective antigens (**Figure 9**). The best protein concentration for discrimination in the case of BPSS1498 seems, therefore, to be between 0.1 and 0.2 µg per dot under the conditions applied. Similar results were observed for BPSL2697, but in contrast to BPSS1498, the background on the diluted spots remained constant and at a low level (**Figure 9**). We measured an increase in specific signal intensities with increased protein concentration per dot and low background in mel (-) sera for almost all other proteins tested. The protein antigens BPSL1763, BPSL2403, BPSL3315, BPSL2925 and BPSS1840 particularly show great promise as serodiagnostic biomarkers giving high and specific signals even at low protein concentrations per dot and high mel (+) to mel (-) ratios.

BPSL1763 is a putative exported chitinase that was detected under almost all stress conditions in the extracellular protein extract indicating general importance for *B. pseudomallei* survival. Its universal expression increases their likelihood of an antibody production within humans and, thereby, a role as a putative potent biomarker. BPSL2403 is a non-hemolytic phospholipase C, which is secreted into the medium via a type 2 secretion system, such as BPSL1763 (58), indicating the importance for environmental survival rather than virulence for both of them. BPSS1840 is a putative N-acetylmuramoyl-L-alanine amidase involved in cell wall metabolism. Hence, it is plausible to assume that it is also present under infection conditions, which could make it a valuable biomarker for melioidosis. BPSL2925 is a glutamate dehydrogenase and important in amino acid metabolism, while the function of BPSL3315 is unknown.

The remaining candidates (BPSS1509, BPSL1510, BPSL1743, BPSS0563, BPSS1103, BPSS1856 and BPSS0860) showed lower signals resulting in lower ratios but might very well serve as valuable biomarkers.

## Discussion

Serologic testing for melioidosis has recently attracted considerable attention (13, 15, 16, 19). Here, the multiplex detection of complementary antibodies can further increase both sensitivity and specificity, and contribute to the reliability of the test, as we could show in our recent studies (13, 19). However, the known cross-reactivity of antibodies to *B. pseudomallei* and other *Burkholderia* species in both melioidosis patients and controls (6, 32, 59) and the potential use of serodiagnostic antigens in vaccines (60) require the detection of further biomarkers with a high specificity and sensitivity for an optimal serological diagnostic of melioidosis in endemic regions. Furthermore, even (multiplex-) assays with the best serodiagnostic antigens give false negative results (15, 16, 20) as there are still individuals who showed no response against any of the antigens (13). Additionally, expanding the repertoire of antigens in multiplex assays that have the ability to detect individual antigens can provide a comprehensive understanding of an individual’s immune response to *B. pseudomallei*. Thus, even if certain antigens are not useful for diagnostic purposes, they could serve as potential biomarkers, for example, regarding immune protection.

Therefore, in this study, we applied a comprehensive immunoproteomic approach to determine melioidosis biomarkers that, as an added benefit, could also be used as (subunit) vaccine candidates. Most studies searching for immunogenic antigens of bacterial pathogens rely on standard cultivation conditions, which do not closely resemble the conditions encountered during infection of the human host (61–63). Invading pathogens normally face massive innate and acquired immune defense, which includes ROS/RNS exposure, acidification, high osmolarity, nutrient/iron limitations and/or hypoxic to anoxic conditions at the site of infection (64–70). Therefore, our goal was to mimic the stress conditions experienced by bacteria in the human host in order to provoke a change in the proteome of *B. pseudomallei* and, thus, increase the pool of antigen candidates that can be screened. It should be noted that *B. pseudomallei* must also survive in (and escape from) environments with osmotic stress (e.g. phagosomes or lymphoid tissues), which is why we chose an elevated salt concentration in our screening of stressors. Furthermore, such conditions (320 mM NaCl), as shown by P. Pumirat and colleagues (71), induce the expression of proteins of the type III secretion system, which are important for intracellular survival.

Since strong immunogenic reactions were not only observed to secreted proteins but also to intracellular proteins such as GroEL or AhpC (28) we analyzed the intracellular as well as the extracellular protein fraction. Protein antigens were identified using IgG detection, which is extensively applied in melioidosis serology (13, 15, 19, 72). Although IgG is likely to be more specific compared to an early IgM response, a limitation of our study is that we cannot completely exclude the possibility that some antigens were not detected because the sera were collected at a time when an IgG class switch of antibodies to a particular antigen had not yet occurred. Future studies using the identified antigens and a large collection of unpooled sera should definitively evaluate the elusive utility of Ig subclasses in the diagnosis of melioidosis by analyzing sensitivity and specificity at the single antigen level and at different time points.

Our strategy was successful, and qualitative and quantitative differences in protein expressions were clearly visible (**Figure 2A** extracellular protein pattern under all conditions tested). The protein fractions of cells exposed to “immune defense-like stressors/conditions” resembling not only ROS (H_2_O_2_), acidification, high osmolarity and anaerobiosis but also nutrient limitation in LB media covered 95% of all antigens identified in this study. Thus, these types of stresses have probably occurred in a similar way in the melioidosis positive donors. Indeed, the function of several antigens detected fit the physiological context under which they were identified, underlining the importance of the proteome shift, which allows the identification of additional otherwise non-expressed proteins. We detected IB signals to the arginine deiminase (BPSL1743) and ornithine carbamoyltransferase (BPSL1744), for example, in protein extracts obtained from bacteria cultivated under anaerobic conditions. Both enzymes are important for the arginine fermentation pathway in many species and are normally expressed under strict anaerobic conditions (73–76). Furthermore, it might show that anaerobic conditions most likely occurred during melioidosis and the arginine fermentation pathway could play an important role for *B. pseudomallei* to gain energy during infections of humans as we see an immune response against these proteins (76). Further examples, pH stress and/or osmolarity stress by NaCl, provoked the secretion of type 2 and 3 secretion system-dependent proteins (71, 77) and we found strong antibody response against BPSS1512 (TssM) and BPSS1529 (BipD), proteins secreted by a type 2 or 3 secretion system, respectively. These proteins are important for the survival inside cells because they are involved in the modulation of cell immunity or the phagosomal escape (78–80), which represent conditions of temporarily increased osmolarity and low pH.

By contrast, only a few immunogenic proteins were detected in cells grown in M9 minimal medium after RNS stress and iron depletion. The M9 medium contained only glucose as a carbon and ammonium chloride as the nitrogen source, which probably allowed only a limited protein expression of many important proteins. At first glance, the limited reaction to iron depletion and RNS stress may appear counterintuitive, given that these stresses are recognized defense mechanisms of the immune system that should trigger an antibody response against stress-specific proteins produced by the bacteria (66, 67, 70). However, it might very well be that the proteins expressed are either low immunogenically or these conditions lead to the expression and/or secretion of small secondary metabolites [as described for iron depletion (81)] which were not detected in IB experiments. In addition, we cannot rule out that these types of host-mediated immune stresses play only a subordinate role during melioidosis in humans resulting in the low antibody response observed against proteins expressed under these conditions. Overall, these results demonstrate the benefit of our approach, which involves the use of various infection-mimicking conditions in conjunction with the extraction of multiple protein fractions. This strategy has enabled us to increase the number of proteins expressed and, consequently, the number of specific IB signals, thereby, facilitating the identification of novel biomarker candidates. This is in agreement with a study of Ooi and colleagues who could show that different growth conditions significantly influence the gene expression profile of *B. pseudomallei* (56).

As 2D IB blots can only resolve protein spots to a certain resolution, we reevaluated the novel biomarker candidates and targets from the literature by dot blot experiments. Indeed, the seroreactivity of some proteins, such as BPSS100 or BPSS144, was not observed in our Dot Blot experiments. A plausible explanation is that their spot overlapped with another protein that gave rise to a strong signal but could not be identified. Moreover, strong cross-reactions obtained with sera from healthy controls gave lastly a low induction rate for these and further candidates, showing the importance of involving an additional validation step, such as our dot plot experiments, in this workflow/protocol. Indeed, the dot blot analysis reconfirmed the serodiagnostic potential of at least five new antigens, which showed induction ratios higher than 10 and have not yet been described as proteins for melioidosis serology. However, it should be noted that a total of 30 proteins including known immunogenic proteins, showed higher induction ratios than the established serodiagnostic marker BPSS1498. It should be emphasized that our five most promising candidates (BPSL1763, BPSL2403, BPSL2925, BPSS1840 and BPSL3315) showed excellent discrimination between positive and negative pooled sera even at low spotting concentrations and a level comparable to known and well-established melioidosis markers, such as BPSS1498 (16, 19), BPSL2697 (13, 28), BPSL2096 (13, 28) and BPSS1529 (82) (**Figure 9**). The latter and other reevaluated antigens once again perfectly demonstrated their suitability as melioidosis biomarkers as described recently and, hence, validate our approach. Furthermore, an enhanced T cell response against BPSL2096 encoding an alkyl hydroperoxide reductase and BPSL2697 encoding GroEL has previously been shown to be associated with survival in melioidosis patients (27). However, some literature targets that have been described as good melioidosis markers (BPSS0908 and BPSS1599) proved to be of limited value in our study (28), which might be due to different experimental setups. This suggests that a comprehensive search for potential antigens should generally involve testing a variety of matrices, techniques and proteins. In conclusion, our approach led to the identification of at least five previously uncharacterized protein antigens. Future studies will have to validate those candidates in multiplex assays for their diagnostic performance on single antigen level and with unpooled sera. Furthermore, our study highlights the significance and efficacy of identifying immunogenic biomarker candidates under nonstandard conditions, and, thus, this approach is likely to be valuable for a wide range of pathogens, whereas the combination of different stressors might also offer a further option for identifying additional biomarkers

## Data availability statement

The raw data supporting the conclusions of this article will be made available by the authors, without undue reservation.

## Ethics statement

The studies involving humans were approved by Faculty of Tropical Medicine, Mahidol University (Submission number TMEC 12–014) Sappasithiprasong Hospital, Ubon Ratchathani (reference 018/2555) Oxford Tropical Research Ethics Committee (reference 64–11). The studies were conducted in accordance with the local legislation and institutional requirements. The human samples used in this study were acquired from primarily isolated as part of your previous study for which ethical approval was obtained. Written informed consent for participation was not required from the participants or the participants’ legal guardians/next of kin in accordance with the national legislation and institutional requirements.

## Author contributions

GW: Data curation, Formal analysis, Investigation, Methodology, Validation, Visualization, Writing – original draft, Writing – review & editing. TS: Conceptualization, Formal analysis, Investigation, Methodology, Validation, Visualization, Writing – original draft. DA: Formal analysis, Methodology, Writing – review & editing. ML: Formal analysis, Methodology, Writing – review & editing. SD: Resources, Writing – review & editing. EF-H: Formal analysis, Methodology, Writing – review & editing. KR: Methodology, Formal analysis, Investigation, Writing – review & editing. AK: Methodology, Writing – review & editing. IS: Conceptualization, Funding acquisition, Investigation, Resources, Supervision, Writing – review & editing. CK: Conceptualization, Data curation, Formal analysis, Investigation, Methodology, Supervision, Validation, Visualization, Writing – original draft, Writing – review & editing.
