# Supplementary material for: Deciphering the human antibody response against Burkholderia pseudomallei during melioidosis using a comprehensive immunoproteome approach
Source: Front Immunol. 2023 Dec 11;14:1294113. doi: 10.3389/fimmu.2023.1294113 (PMC10749318; doi:10.3389/fimmu.2023.1294113)
Supplement: Supplementary file 1 [file Table_1.docx]

Supplementary Table S1. Bacterial strains and plasmids used in this study.

| Strain | Characteristics | Reference |
| --- | --- | --- |
| *Escherichia coli* DH5a | cloning host | Invitrogen, Life technologies, Darmstadt, Germany |
| *E. coli* BL21 (DE3) pLysS | expression strain | Invitrogen, Life technologies, Darmstadt, Germany |
|  |  |  |
| Plasmids |  |  |
| pPR-IBA1 | expression vector, Strep tag | IBA GmbH, Goettingen, Germany |
| pETM-STMUG | expression vector, TEV cleavable Strep tag | Steinmetz Lab |
| pET TrxA-1a | expression vector, TEV cleavable HIS-TRX-tag | European Molecular Biology Laboratory, Heidelberg, Germany |
| pET Z2-1a | expression vector, TEV cleavable HIS-Z2-tag | European Molecular Biology Laboratory, Heidelberg, Germany |
| pET28a | expression vector, HIS-tag | MilliporeSigma (Novagen), Germany |
| pET MBP-1a | expression vector, TEV cleavable HIS-MBP-tag | European Molecular Biology Laboratory, Heidelberg, Germany |
