# Supplementary material for: Deciphering the human antibody response against Burkholderia pseudomallei during melioidosis using a comprehensive immunoproteome approach
Source: Front Immunol. 2023 Dec 11;14:1294113. doi: 10.3389/fimmu.2023.1294113 (PMC10749318; doi:10.3389/fimmu.2023.1294113)
Supplement: Supplementary file 2 [file Table_2.docx]

Supplementary Table S2. Candidates obtained from 2D Western blot analyses and used for dot blot analyses.

| **locus tag** | **function** | **main role** | **sub role** |
| --- | --- | --- | --- |
| BPSL3290 | adenosylhomocysteinase | Amino acid metabolism | Amino acids and amines |
| BPSL0298 | argininosuccinate synthase | Amino acid metabolism | Arginine, glutamate metabolism |
| BPSS0913 | methionine-gamma-lyase | Amino acid metabolism | Cysteine and methionine metabolism |
| BPSS1691 | O-succinylhomoserine sulfhydrylase | Amino acid metabolism | Cysteine and methionine metabolism |
| BPSL2171 | succinyl-diaminopimelate desuccinylase | Amino acid metabolism | Lysine metabolism |
| BPSL2289 | cysteine desulfurase | Biosynthesis of cofactors, prosthetic groups, and carriers | Thiamine and other |
| BPSS1103 | putative periplasmic thiamine-binding protein | Biosynthesis of cofactors, prosthetic groups, and carriers | Thiamine and other |
| BPSL2506 | putative exported transglycosylase | Cell envelope | Biosynthesis and degradation of murein sacculus and peptidoglycan |
| BPSS1840 | putative N-acetylmuramoyl-L-alanine amidase | Cell envelope | Biosynthesis and degradation of murein sacculus and peptidoglycan |
| BPSL3096 | putative bacteriophage-related peptidase | Cell envelope | Other |
| BPSL1801 | putative type-1 fimbrial protein | Cell envelope | Surface structures |
| BPSL2096 | putative hydroperoxide reductase | Cellular processes | Adaptations to atypical conditions |
| BPSL3120 | putative stringent response transcriptional regulator | Cellular processes | Adaptations to atypical conditions |
| BPSL1356 | cell division protein | Cellular processes | Cell division |
| BPSL3021 | cell division protein FtsA | Cellular processes | Cell division |
| BPSS0860 | putative flagellar hook-associated protein | Cellular processes | Chemotaxis and motility |
| BPSL2748 | putative oxidoreductase | Cellular processes | Detoxification |
| BPSL2840 | putative flavohemoglobin-like protein | Cellular processes | Detoxification |
| BPSL2987 | hypothetical | Cellular processes | Detoxification |
| BPSL2403 | phospholipase C | Cellular processes | Inositol phosphate/glycerophospholipid metabolism |
| BPSS0100 | type VI secretion system protein VasD | Cellular processes | Pathogenesis |
| BPSS1395 | type III secretion system protein HrpB | Cellular processes | Pathogenesis |
| BPSS1529 | Invasin D | Cellular processes | Pathogenesis |
| BPSL1743 | arginine deiminase | Energy metabolism | Arginine fermentation |
| BPSL1744 | ornithine carbamoyltransferase, catabolic | Energy metabolism | Arginine fermentation |
| BPSL3396 | ATP synthase beta chain | Energy metabolism | ATP-proton motive force interconversion |
| BPSL1763 | putative exported chitinase | Energy metabolism | Biosynthesis and degradation of polysaccharides |
| BPSL2666 | phosphoglucomutase | Energy metabolism | Carbohydrate metabolism |
| BPSS0144 | putative amylase | Energy metabolism | Carbohydrate metabolism |
| BPSS1571 | probable NADH oxidoreductase | Energy metabolism | Electron transport |
| BPSL2747 | putative glutaryl-CoA dehydrogenase | Energy metabolism | Fatty acid/Amino acid metabolism |
| BPSL2270 | enolase | Energy metabolism | Glycolysis/gluconeogenesis |
| BPSL1955 | 3-oxoacid CoA-transferase subunit A | Energy metabolism | Ketone bodies, valine, leucine, isoleucine and other |
| BPSL1536 | acetoacetyl-CoA reductase | Energy metabolism | Other |
| BPSL2299 | putative dihydrolipoamide dehydrogenase | Energy metabolism | Pyruvate dehydrogenase |
| BPSL2300 | dihydrolipoamide acetyltransferase component of pyruvate dehydrogenase complex | Energy metabolism | Pyruvate dehydrogenase |
| BPSL0960 | putative sulfate adenylyltransferase subunit 1 | Energy metabolism | Sulfur metabolism |
| BPSL0896 | isocitrate dehydrogenase | Energy metabolism | TCA cycle |
| BPSL3056 | acyl carrier protein phosphodiesterase | Fatty acid and phospholipid metabolism | Other |
| BPSL1622 | hypothetical protein | Hypothetical proteins |  |
| BPSL2182 | conserved hypothetical protein | Hypothetical proteins |  |
| BPSL2576 | hypothetical protein | Hypothetical proteins |  |
| BPSL2703 | putative exported protein | Hypothetical proteins |  |
| BPSL2715 | putative lipoprotein | Hypothetical proteins |  |
| BPSL3098 | conserved hypothetical protein | Hypothetical proteins |  |
| BPSS0819 | hypothetical protein | Hypothetical proteins |  |
| BPSS1588 | hypothetical protein | Hypothetical proteins |  |
| BPSL0156 | phage baseplate assembly protein | Mobile and extrachromosomal element functions | Prophage functions |
| BPSL2305 | oligopeptidase A | Protein fate | Degradation of proteins, peptides, and glycopeptides |
| BPSS0563 | putative peptidase | Protein fate | Degradation of proteins, peptides, and glycopeptides |
| BPSS0666 | putative collagenase | Protein fate | Degradation of proteins, peptides, and glycopeptides |
| BPSS0827 | putative collagenase | Protein fate | Degradation of proteins, peptides, and glycopeptides |
| BPSS1992 | putative exported peptidase | Protein fate | Degradation of proteins, peptides, and glycopeptides |
| BPSL1087 | chaperone protein | Protein fate | Protein folding and stabilization |
| BPSL1402 | trigger factor | Protein fate | Protein folding and stabilization |
| BPSL2246 | peptidyl-prolyl cis-trans isomerase B | Protein fate | Protein folding and stabilization |
| BPSL2697 | 60 kDa chaperonin | Protein fate | Protein folding and stabilization |
| BPSL2827 | putative DnaK chaperone protein | Protein fate | Protein folding and stabilization |
| BPSL2515 | 30s ribosomal protein s1 | Protein synthesis | Ribosomal proteins: synthesis and modification |
| BPSL3188 | 30S ribosomal protein S4 | Protein synthesis | Ribosomal proteins: synthesis and modification |
| BPSL2158 | elongation factor TS | Protein synthesis | Translation factors |
| BPSL3215 | elongation factor Tu | Protein synthesis | Translation factors |
| BPSL3228 | elongation factor Tu | Protein synthesis | Translation factors |
|  |  |  |  |

***^a^*** Locus name, function, main role and sub role were used from *B. pseudomallei* strain K96243 and obtained from Kyoto Encyclopedia of Genes and Genomes ([www.genome.jp/kegg/](http://www.genome.jp/kegg/))
