# Supplementary material for: Deciphering the human antibody response against Burkholderia pseudomallei during melioidosis using a comprehensive immunoproteome approach
Source: Front Immunol. 2023 Dec 11;14:1294113. doi: 10.3389/fimmu.2023.1294113 (PMC10749318; doi:10.3389/fimmu.2023.1294113)
Supplement: Supplementary file 3 [file Table_3.docx]

Supplementary Table 3. Literature targets, virulence factors and other additional proteins used for dot blot analyses to confirm serodiagnostic potential.

| **locus tag*^a^*** | **function*^a^*** | **main role*^a^*** | **sub role*^a^*** | **source*^b^*** |
| --- | --- | --- | --- | --- |
| BPSL3168 | 3-dehydroquinate synthase | Amino acid biosynthesis | Aromatic amino acid family | [1] |
| BPSL1962 | chorismate synthase | Amino acid biosynthesis | Aromatic amino acid family | [2] |
| BPSL2925 | glutamate dehydrogenase | Amino acid biosynthesis | Glutamate family | available in the lab |
| BPSL3133 | imidazole glycerol phosphate synthase subunit HisF | Amino acid biosynthesis | Histidine family | [3] |
| BPSL1196 | acetolactate synthase isozyme III large subunit | Amino acid biosynthesis | Pyruvate family | [4] |
| BPSL2825 | para-aminobenzoate synthetase / 4-amino-4-deoxychorismate lyase | Biosynthesis of cofactors, prosthetic groups, and carriers | Folic acid | [3] |
| BPSL0413 | lipoate-protein ligase B | Biosynthesis of cofactors, prosthetic groups, and carriers | Lipoic acid metabolism | [5] |
| BPSL0919 | LytB protein | Biosynthesis of cofactors, prosthetic groups, and carriers | Other | [6] |
| BPSL0999 | putative OmpA family transmembrane protein | Cell envelope | Other | [7] |
| BPSL2765 | putative peptidoglycan-associated lipoprotein precursor | Cell envelope | Other | [6-8] |
| BPSL1528 | conserved hypothetical protein | Cell envelope | Other | [3] |
| BPSS1740 | lipase chaperone | Cell envelope | Surface structures | [5] |
| BPSL3170 | hypothetical protein | Cell envelope | Surface structures | [9] |
| BPSS0908 | surface-exposed protein | Cellular processes | unknown | [7, 10] |
| BPSL0225 | putative flagellar hook-length control protein | Cellular processes | Chemotaxis and motility | available in the lab |
| BPSS1498 | type VI secretion system secreted protein Hcp | Cellular processes | Pathogenesis | [8, 11] |
| BPSS1539 | hypothetical protein (type VI secretion system) | Cellular processes | Pathogenesis | [3] |
| BPSS1599 | type IV pilus biosynthesis protein | Cellular processes | Pathogenesis | [7, 12, 13] |
| BPSS1524 | intercellular spread protein | Cellular processes | Pathogenesis | [8, 14] |
| BPSS1531 | putative cell invasion protein | Cellular processes | Pathogenesis | [7, 14, 15] |
| BPSS1548 | Type III secretion system protein | Cellular processes | Pathogenesis | [14] |
| BPSS1856 | phosphotransferase | Central intermediary metabolism | Unknown substrate | [16] |
| BPSS0897 | putative short chain dehydrogenase | Central intermediary metabolism | Unknown substrate | [16] |
| BPSL1452 | glyoxylate carboligase | Central intermediary metabolism | Unknown substrate | available in the lab |
| BPSS0467 | putrescine ABC transport system, binding exported protein | Transport and binding proteins | Unknown substrate | [17, 18] |
| BPSL2504 | putative hydrolase | Central intermediary metabolism | Unknown substrate | [19] |
| BPSS1757 | conserved hypothetical protein | Central intermediary metabolism | Unknown substrate | [16] |
| BPSL3315 | putative Cof family hydrolase | Central intermediary metabolism | Unknown substrate | available in the lab |
| BPSS0680 | isoquinoline 1-oxidoreductase alpha subunit | Central intermediary metabolism | Unknown substrate | available in the lab |
| BPSS0620 | 3-hydroxyisobutyrate dehydrogenase | Energy metabolism | Amino acids and amines | available in the lab |
| BPSS1904 | benzoate 1,2-dioxygenase beta subunit | Energy metabolism | Electron transport | [16] |
| BPSL1907 | dihydrolipoamide dehydrogenase | Energy metabolism | Pyruvate dehydrogenase | available in the lab |
| BPSL0853 | putative cyclopropane-fatty-acyl-phospholipid synthase | Fatty acid and phospholipid metabolism | Phospholipid metabolism | available in the lab |
| BPSL1050 | hypothetical protein | Hypothetical proteins |  | [6] |
| BPSS1509 | conserved hypothetical protein | Hypothetical proteins |  | [3] |
| BPSS1492 | hypothetical protein | Hypothetical proteins |  | [7, 8, 12] |
| BPSL2818 | putative phosphoribosylformyl-glycinamidine cyclo-ligase | Purines, pyrimidines, nucleosides, and nucleotides | Purine ribonucleotide biosynthesis | [3] |
| BPSL1510 | nucleoside diphosphate kinase | Purines, pyrimidines, nucleosides, and nucleotides | Purine/Pyrimidine metabolism | available in the lab |
| GFP | green fluorescent protein |  | negativ control | available in the lab |

***^a^*** Locus name, function, main role and sub role were used from *B. pseudomallei* strain K96243 and obtained from Kyoto Encyclopedia of Genes and Genomes ([www.genome.jp/kegg/](http://www.genome.jp/kegg/))

***^b^***  Proteins were chosen from literature or were already available in the laboratory (lab).

1. Cuccui J, Easton A, Chu KK, Bancroft GJ, Oyston PC, Titball RW, et al. Development of signature-tagged mutagenesis in Burkholderia pseudomallei to identify genes important in survival and pathogenesis. Infect Immun (2007) 75(3):1186-95. doi: 10.1128/IAI.01240-06. PubMed PMID: 17189432; PubMed Central PMCID: PMC1828585.

2. Srilunchang T, Proungvitaya T, Wongratanacheewin S, Strugnell R, Homchampa P. Construction and characterization of an unmarked aroC deletion mutant of Burkholderia pseudomallei strain A2. Southeast Asian J Trop Med Public Health (2009) 40(1):123-30. PubMed PMID: 19323044.

3. Pilatz S, Breitbach K, Hein N, Fehlhaber B, Schulze J, Brenneke B, et al. Identification of Burkholderia pseudomallei genes required for the intracellular life cycle and in vivo virulence. Infect Immun (2006) 74(6):3576-86. doi: 10.1128/IAI.01262-05. PubMed PMID: 16714590; PubMed Central PMCID: PMC1479254.

4. Haque A, Chu K, Easton A, Stevens MP, Galyov EE, Atkins T, et al. A live experimental vaccine against Burkholderia pseudomallei elicits CD4+ T cell-mediated immunity, priming T cells specific for 2 type III secretion system proteins. J Infect Dis (2006) 194(9):1241-8. doi: 10.1086/508217. PubMed PMID: 17041850.

5. Breitbach K, Kohler J, Steinmetz I. Induction of protective immunity against Burkholderia pseudomallei using attenuated mutants with defects in the intracellular life cycle. Trans R Soc Trop Med Hyg (2008) 102 Suppl 1:S89-94. doi: 10.1016/S0035-9203(08)70022-1. PubMed PMID: 19121696.

6. Peri C, Gori A, Gagni P, Sola L, Girelli D, Sottotetti S, et al. Evolving serodiagnostics by rationally designed peptide arrays: the Burkholderia paradigm in Cystic Fibrosis. Sci Rep (2016) 6:32873. doi: 10.1038/srep32873. PubMed PMID: 27615705; PubMed Central PMCID: PMC5018727.

7. Felgner PL, Kayala MA, Vigil A, Burk C, Nakajima-Sasaki R, Pablo J, et al. A Burkholderia pseudomallei protein microarray reveals serodiagnostic and cross-reactive antigens. Proc Natl Acad Sci U S A (2009) 106(32):13499-504. doi: 10.1073/pnas.0812080106. PubMed PMID: 19666533; PubMed Central PMCID: PMC2717108.

8. Titball RW, Burtnick MN, Bancroft GJ, Brett P. Burkholderia pseudomallei and Burkholderia mallei vaccines: Are we close to clinical trials? Vaccine (2017) 35(44):5981-9. doi: 10.1016/j.vaccine.2017.03.022. PubMed PMID: 28336210.

9. Essex-Lopresti AE, Boddey JA, Thomas R, Smith MP, Hartley MG, Atkins T, et al. A type IV pilin, PilA, contributes to adherence of Burkholderia pseudomallei and virulence in vivo. Infect Immun (2005) 73(2):1260-4. doi: 10.1128/IAI.73.2.1260-1264.2005. PubMed PMID: 15664977; PubMed Central PMCID: PMC547011.

10. Lafontaine ER, Zimmerman SM, Shaffer TL, Michel F, Gao X, Hogan RJ. Use of a safe, reproducible, and rapid aerosol delivery method to study infection by Burkholderia pseudomallei and Burkholderia mallei in mice. PLoS One (2013;) 8(10):e76804. doi: 10.1371/journal.pone.0076804. PubMed PMID: 24098563; PubMed Central PMCID: PMC3788738.

11. Phokrai P, Karoonboonyanan W, Thanapattarapairoj N, Promkong C, Dulsuk A, Koosakulnirand S, et al. A rapid immunochromatography test based on Hcp1 is a potential point-of-care test for serological diagnosis of melioidosis. J Clin Microbiol (2018) 56(8):e00346-18. doi: 10.1128/JCM.00346-18. PubMed PMID: 29848565; PubMed Central PMCID: PMC6062804.

12. Suwannasaen D, Mahawantung J, Chaowagul W, Limmathurotsakul D, Felgner PL, Davies H, et al. Human immune responses to Burkholderia pseudomallei characterized by protein microarray analysis. J Infect Dis (2011) 203(7):1002-11. doi: 10.1093/infdis/jiq142. PubMed PMID: 21300673; PubMed Central PMCID: PMC3068035.

13. Varga JJ, Vigil A, DeShazer D, Waag DM, Felgner P, Goldberg JB. Distinct human antibody response to the biological warfare agent Burkholderia mallei. Virulence (2012( 3(6):510-4. doi: 10.4161/viru.22056. PubMed PMID: 23076276; PubMed Central PMCID: PMC3524150.

14. Vander Broek CW, Stevens JM. Type III secretion in the melioidosis pathogen Burkholderia pseudomallei. Front Cell Infect Microbiol (2017) 7:255. doi: 10.3389/fcimb.2017.00255. PubMed PMID: 28664152; PubMed Central PMCID: PMC5471309.

15. Druar C, Yu F, Barnes JL, Okinaka RT, Chantratita N, Beg S, et al. Evaluating Burkholderia pseudomallei Bip proteins as vaccines and Bip antibodies as detection agents. FEMS Immunol Med Microbiol (2008) 52(1):78-87. doi: 10.1111/j.1574-695X.2007.00345.x. PubMed PMID: 17995960.

16. Puah SM, Puthucheary SD, Chua KH. Potential immunogenic polypeptides of Burkholderia pseudomallei identified by shotgun expression library and evaluation of their efficacy for serodiagnosis of melioidosis. Int J Med Sci (2013) 10(5):539-47. doi: 10.7150/ijms.5516. PubMed PMID: 23532805; PubMed Central PMCID: PMC3607239.

17. Harland DN, Chu K, Haque A, Nelson M, Walker NJ, Sarkar-Tyson M, et al. Identification of a LolC homologue in Burkholderia pseudomallei, a novel protective antigen for melioidosis. Infect Immun (2007) 75(8):4173-80. doi: 10.1128/IAI.00404-07. PubMed PMID: 17517877; PubMed Central PMCID: PMC1951986.

18. Matthysse AG, Yarnall HA, Young N. Requirement for genes with homology to ABC transport systems for attachment and virulence of Agrobacterium tumefaciens. J Bacteriol (1996) 178(17):5302-8. doi: 10.1128/jb.178.17.5302-5308.1996. PubMed PMID: 8752352; PubMed Central PMCID: PMC178331.

19. Dunachie SJ, Jenjaroen K, Reynolds CJ, Quigley KJ, Sergeant R, Sumonwiriya M, et al. Infection with Burkholderia pseudomallei – immune correlates of survival in acute melioidosis. Sci Rep (2017) 7(1):12143. doi: 10.1038/s41598-017-12331-5. PubMed PMID: 28939855; PubMed Central PMCID: PMC5610189.
